# Automatic Classification of Nodules from 2D Ultrasound Images Using Deep Learning Networks

**DOI:** 10.3390/jimaging10080203

**Published:** 2024-08-22

**Authors:** Tewele W. Tareke, Sarah Leclerc, Catherine Vuillemin, Perrine Buffier, Elodie Crevisy, Amandine Nguyen, Marie-Paule Monnier Meteau, Pauline Legris, Serge Angiolini, Alain Lalande

**Affiliations:** 1 ICMUB Laboratory, UMR CNRS 6302, University of Burgundy, 7 Bld Jeanne d’Arc, 21000 Dijon, France; tewele-weletnsea.tareke@u-bourgogne.fr (T.W.T.); sarah.leclerc@u-bourgogne.fr (S.L.); 2 Medical Imaging Department, Hospital of Bastia, 20600 Bastia, France; cath.vuillemin@hotmail.fr (C.V.); angiolini.serge@gmail.com (S.A.); 3 Department of Endocrinology-Diabetology, University Hospital, 21000 Dijon, France; perrine.buffier@chu-dijon.fr (P.B.); elodie.crevisy@chu-dijon.fr (E.C.); amandine.nguyen@chu-dijon.fr (A.N.); marie-paule.monnier@chu-dijon.fr (M.-P.M.M.); pauline.legris@chu-dijon.fr (P.L.); 4 Department of Medical Imaging, University Hospital of Dijon, 21000 Dijon, France

**Keywords:** Bethesda score, classification, DenseNet, fine needle aspiration, Grad-CAM, thyroid nodule, ultrasound image

## Abstract

Objective: In clinical practice, thyroid nodules are typically visually evaluated by expert physicians using 2D ultrasound images. Based on their assessment, a fine needle aspiration (FNA) may be recommended. However, visually classifying thyroid nodules from ultrasound images may lead to unnecessary fine needle aspirations for patients. The aim of this study is to develop an automatic thyroid ultrasound image classification system to prevent unnecessary FNAs. Methods: An automatic computer-aided artificial intelligence system is proposed for classifying thyroid nodules using a fine-tuned deep learning model based on the DenseNet architecture, which incorporates an attention module. The dataset comprises 591 thyroid nodule images categorized based on the Bethesda score. Thyroid nodules are classified as either requiring FNA or not. The challenges encountered in this task include managing variability in image quality, addressing the presence of artifacts in ultrasound image datasets, tackling class imbalance, and ensuring model interpretability. We employed techniques such as data augmentation, class weighting, and gradient-weighted class activation maps (Grad-CAM) to enhance model performance and provide insights into decision making. Results: Our approach achieved excellent results with an average accuracy of 0.94, F1-score of 0.93, and sensitivity of 0.96. The use of Grad-CAM gives insights on the decision making and then reinforce the reliability of the binary classification for the end-user perspective. Conclusions: We propose a deep learning architecture that effectively classifies thyroid nodules as requiring FNA or not from ultrasound images. Despite challenges related to image variability, class imbalance, and interpretability, our method demonstrated a high classification accuracy with minimal false negatives, showing its potential to reduce unnecessary FNAs in clinical settings.

## 1. Introduction

Thyroid nodules, often benign, can occur in the thyroid gland, regulating metabolism. However, some can be cancerous, leading to mortality [1]. Fine needle aspiration (FNA) biopsy helps determine malignancy risk, guiding treatment decisions, despite its invasive nature. Thyroid ultrasonography offers advantages for the evaluation of the thyroid gland such as noninvasiveness, accessibility, and cost-effectiveness. However, there exists inter-observer variability in interpreting images of thyroid nodules. Several ultrasound features have been identified as associated with an increased risk of malignancy, including marked hypo-echogenicity, large size, extra-thyroidal extension, non-oval shape, irregular margins, and the presence of micro-calcifications. The European-Thyroid Imaging Reporting and Data System (EU-TIRADS) was established to standardize thyroid ultrasonography analysis across Europe and stratify risk based on these features [2,3].

Conversely, the Bethesda cytological classification from fine needle aspiration (FNA) is an international classification that standardizes the diagnostic criteria from the cytopunction of the thyroid nodules. It identifies six cytological categories and provides recommendations for each category: clinical follow-up, repeat puncture, lobectomy, or thyroidectomy. The Bethesda cytological system is considered as the main criterion to make the decision to perform surgery [4]. A Bethesda score of II represents a class where FNA is not required. A score of I is interpreted as non-diagnostic, necessitating the repetition of FNA, often due to insufficient thyroid cells. Bethesda scores from III to VI are labeled as atypia of undetermined significance, follicular neoplasm, suspicious for malignancy, or malignant, respectively, indicating that FNA is required. Indeed, thyroid fine-needle-aspiration biopsy is recommended for nodules based on the EU-TIRADS score and size criteria [5]. According to these guidelines, nodules require monitoring if they fall under specific categories: EU-TIRADS 2, EU-TIRADS 3 with a size of ≤20 mm, EU-TIRADS 4 with a size of ≤ 15 mm, and EU-TIRADS 5 with a size of ≤10 mm. Conversely, nodules necessitate FNA if they are categorized as EU-TIRADS 3 with a size of ≥20 mm, EU-TIRADS 4 with a size of ≥15 mm, and EU-TIRADS 5 with a size of ≥10 mm. However, from the EU-TIRADS criteria of image analysis, a significant number of benign nodules result in unnecessary FNAs [6].

Ultrasound imaging is an easy and accurate method for the analysis of thyroid nodules. However, the high variability of the cases and the low image quality are a continual challenge for nodule classification. Detection of thyroid nodules using ultrasound imaging has significantly increased over the past few years, with many more nodules being incidentally detected [7] and requiring visual analysis. Elastography is a non-invasive imaging technique that measures tissue stiffness by evaluating the response of tissues to applied mechanical stress. This modality provides crucial information about tissue elasticity, which is essential for diagnosing various conditions, including tumors. The integration of machine learning with elastography represents a significant advancement in medical imaging and classification. By leveraging machine learning algorithms, elastography can substantially enhance the accuracy and efficiency of tissue characterization, as demonstrated in the study by Mao et al. [8]. This research illustrates how machine-learning-based elastography can effectively assist in identifying endocrine tumors.

In recent years, a few machine learning methods have been proposed to identify the malignancy risk of nodules, whatever the image modality. Peng et al. [9] investigated the feasibility of applying the first-order texture features to diagnose thyroid nodules from computed tomographic images (CT). Chi et al. [10] presented a CAD system to classify a thyroid nodule as malignant or not from ultrasound images. A comparative analysis of various machine learning algorithms, including decision trees, random forests, KNN, and artificial neural networks, was conducted using the University of California, Irvine (UCI) Repository dataset (https://archive.ics.uci.edu/, accessed on 10 June 2021), as detailed in a study by Alyas et al. [11]. The method demonstrated a sensitivity of 0.94. However, there is potential for further improvement.

Deep learning was recently established as a promising tool to improve cancer detection. Zhu et al. [12] proposed an image augmentation method using a convolutional network for thyroid nodule classification via transfer learning. Buda et al. [7] recently tackled the classification problem from ultrasound images using a deep learning algorithm to provide management recommendations thanks to a stratification of the risk level for thyroid nodules. The used networks were a Faster R-CNN network to localize the region of interest and then a multi-task CNN to predict the risk of malignancy. Nguyen et al. [13] proposed an ultrasound-image-based diagnosis method for malignant thyroid nodules using artificial intelligence based on the analysis in both the spatial and frequency domains. A weighted binary cross-entropy loss function was used for training deep convolutional neural networks to reduce the effects of unbalanced training samples of the target classes in the training data. The study by Wu et al. [14] was based on a radial basis function (RBF)–neural network method used as a classifier. A new fine-tuned deep learning model for classifying thyroid nodules in ultrasonography, introduced by Kwon et al. [15], achieved an accuracy of 0.81 and a sensitivity of 0.92. Li et al. [16] developed an end-to-end thyroid nodule automatic recognition and classification system for diagnosing thyroid diseases. U-Net was used to detect the tumor, and a convolutional neural network fusion (CNN-fusion) was employed to classify the thyroid nodule. These previous studies primarily addressed classifying and stratifying thyroid nodules from ultrasound images, referencing the TIRADS ranking. The objective of our study uniquely aims to automatically predict unnecessary fine needle aspirations (FNAs), crucially saving clinician time and reducing patient stress. Thus, our focus lies in predicting FNA necessity, with the Bethesda score serving as the ground truth, diverging from the reliance on TIRADS. Our objective is strictly limited to this classification, offering significant assistance to both patients and clinicians, without extending to estimate the nodule’s malignancy, as it falls beyond the scope of our study. We propose a computer-aided diagnosis (CAD) system based on a deep convolutional neural network to predict whether a thyroid nodule should undergo a fine needle aspiration or not, without making the segmentation of the nodule on the ultrasound image. Overall, automatic thyroid classification offers the potential for faster, more consistent, and reduced inter-observer variability seen among different sonographers, leading to more accurate diagnosis. This ultimately improves patient outcomes and optimizes healthcare workflows. This network is based on the DenseNet deep learning architecture [17], in which attention modules were integrated to focus the processing in the area of interest inside the image. Our main contributions are as follows: 1. We developed a computer-aided diagnosis system for classifying thyroid nodules using the DenseNet architecture, which effectively reduces unnecessary fine needle aspirations. 2. Dual attention modules were integrated into the DenseNet121 deep learning architecture, leading to substantial improvements in the accuracy of thyroid nodule classification on a limited dataset. 3. We demonstrated the interpretability of the classification by utilizing heat maps derived from the Grad-CAM algorithm, specifically to improve the reliability of determining whether fine needle aspiration is needed. The novelty of our work lies in the development of a system that integrates dual attention modules within the DenseNet121 architecture, combined with the use of Grad-CAM to enhance the classification of thyroid nodules, aiding in the determination of the need for fine needle aspiration. By sequentially incorporating channel and spatial attention mechanisms, our approach refines intermediate network features, resulting in more focused and effective classifications. The Grad-CAM heat maps further enhance the system’s reliability by visually validating the regions influencing the decision, thereby improving the model’s interpretability.

In the following sections, this paper explores the methodology used to classify whether nodules require fine needle aspiration, providing a detailed explanation of the dataset utilized in the classification task and the deep learning techniques employed. Section 2 covers data collection and preprocessing methods, as well as the architecture of the neural networks and the attention mechanisms used in this study. Section 3 presents the experimental results and their implications. Section 4 discusses the results, compares our study with related previous work, and provides detailed directions for future development of the proposed system. Finally, Section 5 presents a comprehensive conclusion, summarizing the findings and insights derived from this research.

## 2. Material and Methods

### 2.1. Dataset

For our study, 591 ultrasound (US) images of thyroid nodules were acquired from the Aixplorer and Canon systems. In our research, we used the Aixplorer Ultrasound System (Supersonic Imagine, Aix-en-Provence, France) and the Canon Aplio i800 (Canon Medical Systems, Otawara, Japan) for ultrasound image acquisition. The Aixplorer system was equipped with the SL15-4 linear array transducer, which offers a frequency range of 4 to 15 MHz. The Canon Aplio i800 system was paired with a PVI-475BX transducer, designed for high-resolution imaging. The dimensions of the images from the Aixplorer and Canon systems were 1440 × 1080 and 1280 × 960, respectively. Each nodule has two image orientations or views, as both sagittal and axial views were obtained for each case. Consequently, both orientations were considered during the training of the deep learning model. Of the 591 images, 216 came from the University Hospital of Dijon (France) and 314 from the Hospital of Bastia (France). Based on expert recommendations from the US images, fine needle aspiration (FNA) was performed for all cases. According to the Bethesda score, 198 images were labeled as positive thyroid nodules (requiring FNA), while 393 images were labeled as negative thyroid nodules (not requiring FNA) [18]. The ground truths for our thyroid nodule classification were established through a careful process involving expert radiologists. Each ultrasound image was reviewed and annotated by multiple experienced radiologists to ensure a high level of agreement and accuracy. To further validate the ground truth, we conducted a cross-validation study with an independent set of radiologists who verified the annotations. This multi-rater approach helps to minimize individual biases and errors, ensuring that the ground truth annotations are robust and reliable. Our dataset includes Bethesda categories II, III, IV, V, and VI. The Bethesda I category was excluded from our image dataset because the cytology result is non-diagnostic or unsatisfactory (42 cases classified as Bethesda I were excluded). Out of the resulting 591 ultrasound images, 527 were used for training and validating the model, while 64 images were carefully selected from both centers for the test set. These images were chosen to ensure that the test set was representative of both hospitals, systems, and classes. Table 1 presents a summary of the dataset we used for developing the AI system. To balance the training dataset, 61 public additional thyroid images of nodules established as malignant were also included (Aixplorer system) (https://www.kaggle.com/datasets/dasmehdixtr/ddti-thyroid-ultrasound-images, accessed on 7 June 2022). This public dataset was used only for the training of the neural network. This public dataset includes only Bethesda categories III and IV. Due to the limited size of the dataset, separate training and validation processes on the public dataset were not conducted. The optimal setup we discovered for constructing this model was a split ratio of 70% for training, 20% for validation, and 10% for testing, utilizing a mixed cohort. The ground truth information was extracted from annotated datasets where each sample is labeled as either positive cases (thyroid nodules requiring FNA) or negative (thyroid nodules not requiring FNA). This labeling process involves expert radiologists who review and annotate each sample based on clinical criteria and imaging characteristics.

### 2.2. Pre-Processing

**Figure 1:**
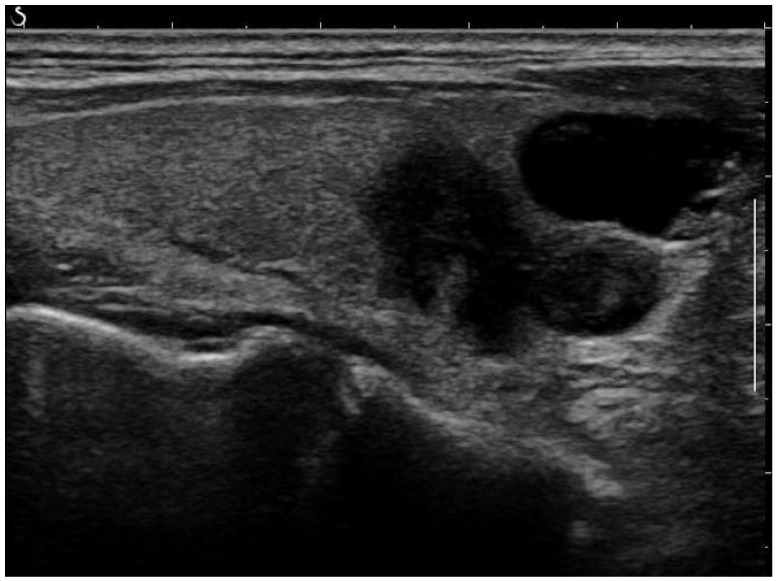
Raw ultrasound image of the thyroid nodule, with noise and artifacts covering part of the textures.

The acquisition settings varied due to differing clinical practices across the hospitals. For our study and analysis, we used retrospective data collected from these diverse settings. Raw images, as in the example shown in Figure 1, were pre-processed to reduce noise and enhance the quality of the input data. Several commonly used image enhancement techniques were performed as follows:

Normalization: Due to the fact that deep learning algorithms are biased towards numerically large values, standardizing the images from different sources to the same scale is essential. All images were normalized to have zero mean and unit variance.

Cropping and resizing: The input images were cropped and resized to have the same image size and resolution.

Histogram Equalization: This process is used to increase the global contrast of the images, especially when the image is represented by a narrow range of intensity values. It is accomplished by spreading out the most frequent intensity values [19].

**Figure 2:**
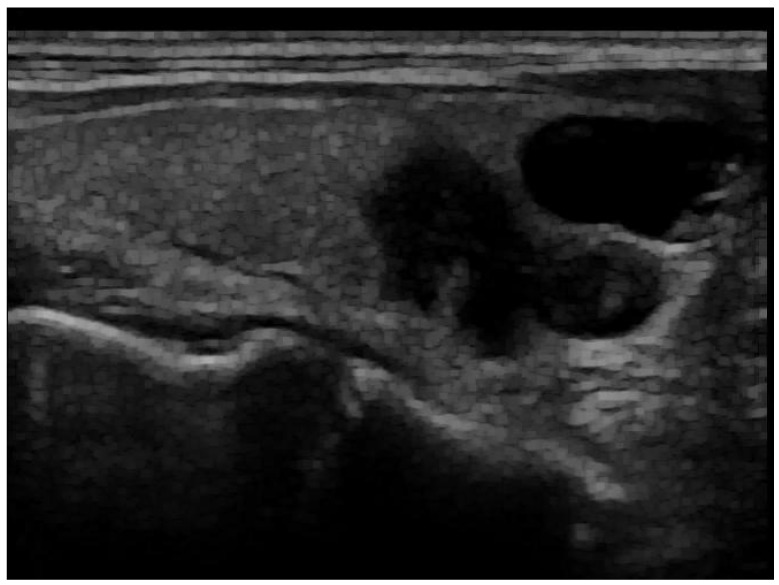
Post-processed image after artefact removal and histogram equalization.

Removing Artefacts: Most of the artefacts are small white lines overlayed on the image. The objective of this process is to discard them while keeping the essential information present in the images. An anchor point (x, y) was used to automatically create a bounding box around artefacts. This box, defined by its width, height, and 4-connectivity method, helped to concentrate the processing around the artefacts. Without this step, there was a risk of inadvertently removing parts of the image that did not contain artefacts. Following this, morphological opening was applied to remove the small white thin lines corresponding to scale for annotation on the images while preserving the shape and size of larger objects in the image. A small structuring element was used to maintain the texture information. An example of a fully post-processed image can be seen in Figure 2.

### 2.3. Data Augmentation

After the images were acquired and cleaned, an AI system was utilized to classify the nodules as requiring FNA or not. A common limitation of using deep learning in medical imaging is the scarcity of the data required for the training step of the neural network. One big challenge of this research work was, therefore, to overcome the limited dataset size by generating synthetic images. To this end, we artificially augmented the number of images in the dataset. At every iteration of the training model, a batch size of transformed images was generated with different parameters including shifting, rotation, flipping, shearing, contrast variability, brightness range, sample and feature-wise standardization, fill mode, and zero component analysis-epsilon. Each generated image is randomly different from the original in certain aspects depending on the augmentation techniques. Therefore, additional images were dynamically generated from the existing dataset on the fly as the model was being trained. The size of the augmented dataset depends on the number of original samples, the augmentation rate, and the number of epochs. The augmentation rate refers to the extent to which data augmentation is applied to the dataset. For our training, we used 527 original samples, with an augmentation rate of 8 for each of the 150 epochs. Consequently, the model was trained on 527×150×8 samples, resulting in a total of 631,800 samples seen by the model during training.

### 2.4. Automatic Classification of Thyroid Nodules

**Figure 3:**
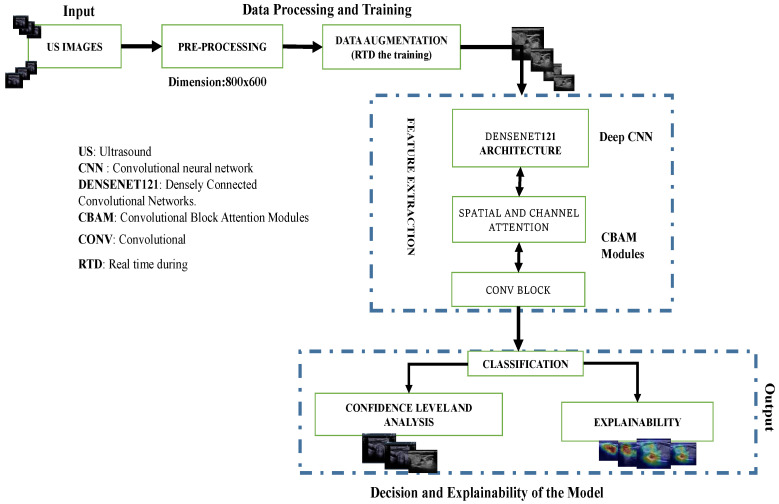
Overview of the proposed pipeline: Deep convolutional neural network (DCNN) model for thyroid nodule image classification. Data augmentation was only applied during training while confidence level analysis and explainability were only conducted during the testing phase.

The overall pipeline is depicted in Figure 3. It consists of pre-processing, data augmentation for the training part, and feature extraction using a pre-trained DenseNet121 network with attention modules to extract the most important information from the final convolutional blocks to perform binary classification of the US images. It also includes the prediction of nodule risk level and post hoc explainability of the process.

**Figure 4:**
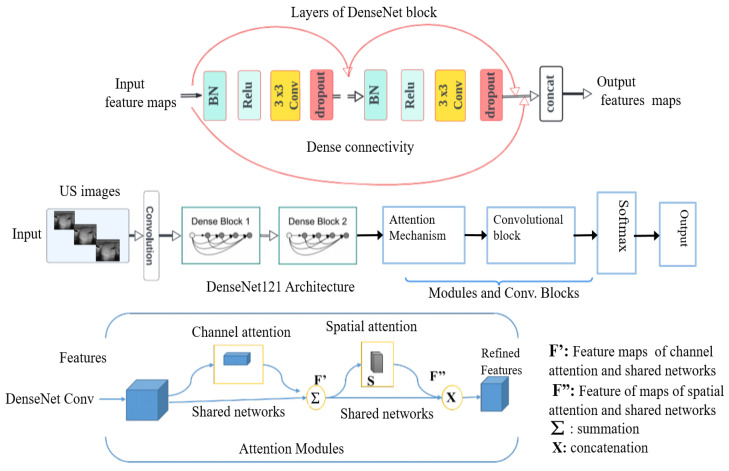
Block diagram of DenseNet121 architecture with incorporated modules, in particular, attention modules.

Deep Convolutional Neural Networks (DCNNs): We studied various convolutional neural networks (CNNs) by selecting both basic and advanced models for comparative analysis. This included simple convolutional neural networks (CNNs) as well as pre-trained models such as VGG16, EfficientNet-B0, ResNet18, InceptionV3, and DenseNet121. These models were pre-trained on ImageNet, a large-scale dataset with over 1 million labeled images across 1000 categories. By covering a spectrum from basic to advanced deep learning models, we utilized a range of techniques trained on the extensive ImageNet dataset to leverage rich and generalized feature representations applicable to our task. This approach effectively addresses the challenge of limited datasets for training from scratch. Initially, we implemented and trained a simple CNN, consisting of 25 layers with a softmax activation function, from scratch on our dataset. Subsequently, we fine-tuned well-established pre-trained deep CNN architectures for our task through transfer learning. ResNet18, a deep convolutional neural network with 72 layers including 18 deep residual layers, was one of the models used [20]. EfficientNet-B0 is a deep learning architecture that was introduced in 2019 [21]. The key innovation of EfficientNet is its use of a compound scaling method that uniformly scales network width, depth, and resolution using a simple yet highly effective formula. VGG16 is a convolutional neural network architecture that was developed by the Visual Geometry Group at the University of Oxford and introduced in 2014 [22]. It is characterized by its simplicity and depth, consisting of 16 weight layers including 13 convolutional layers followed by 3 fully connected layers. InceptionV3 [23] is a highly optimized convolutional neural network architecture that uses asymmetric convolutions, such as 1 × 7 followed by 7 × 1, to approximate larger convolutions. This significantly reduces the number of parameters and computational expense. DenseNet121 network is composed of 427 layers with 120 convolutions and 4 average pooling layers [24]. DenseNet121 is efficient for classification tasks because of its multiple skip connections, which allows better transmission of features across the network and addresses the vanishing and exploding gradients’ problems via inter-layer connection [17]. Unlike residual neural networks (such as ResNet18), the feature maps from previous layers are concatenated and not summed. As a first result of the fine-tuned models, the DenseNet121 architecture outperformed the other studied deep learning networks on our dataset of 591 US images. Therefore, we kept this architecture as a new baseline for the nodule classification. Our proposed method is built upon the DenseNet121 network, incorporating dual attention modules [25] at the output level of the DenseNet121 convolutional blocks. These modules are then connected to the final added convolution block (Figure 4).

The distinctiveness of our proposed approach lies in the specific design and integration of attention modules within the DenseNet121 architecture to enhance feature extraction and classification performance, while many existing methods use attention mechanisms to extract discriminative features, our approach introduces a dual attention mechanism that refines features in the intermediate part of the network. By incorporating both channel attention modules (CAM) and spatial attention modules (SAM) sequentially, our method significantly improves the network’s ability to focus on crucial features and discard irrelevant information.

The combination of channel and spatial attention mechanisms can be used to extract both discriminative features and intermediate features. Tao et al. [26] integrated these modules with DenseNet121 and fine-tuned the network for medical image classification, resulting in enhanced model performance. The purpose of the dual attention modules in this approach is to extract discriminative feature representations for the task of estimating the smoke density of smoky vehicle rears. Similarly, other studies [27,28] have integrated dual attention modules with DenseNet121 to extract discriminative features that are most relevant for distinguishing different classes. Unlike the aforementioned studies, the primary purpose of our proposed dual attention module is to extract crucial intermediate features, refine relevant feature maps, and forward these enhanced features to the subsequent block in our pipeline. We utilize two convolutional attention modules arranged sequentially (Figure 4). The first module, known as the channel attention module, enhances inter-channel relationships in feature maps by scaling them with coefficients learned from max-pooling and average-pooling. This is expressed by the following formula:

(1) $$\begin{array}{c} F^{\prime }=\sigma \left(\mathrm{MLP}\left(\mathrm{AvgPool}\left(F\right)\right)\right)+\sigma \left(\mathrm{MLP}\left(\mathrm{MaxPool}\left(F\right)\right)\right) \end{array}$$

where:

- F is the input feature maps;
- σ represents the sigmoid activation function;
- MLP denotes a multi-layer perceptron;
- [+] denotes summation.

The second module, the spatial attention module, creates an attention map by leveraging inter-spatial relationships. It multiplies feature maps with a heatmap derived from concatenated average-pooling and max-pooling features. Unlike the channel attention module, the spatial attention module focuses on locations abundant in information rather than weighting the entire feature maps. Mathematically, spatial attention is expressed as follows:

(2) $$\begin{array}{c} F^{\prime \prime }=\sigma \left(f^{5\times 5}\left([\mathrm{AvgPool}\left(F^{\prime }\right)\;x\;\mathrm{MaxPool}\left(F^{\prime }\right)]\right)\right) \end{array}$$

where:

- $f^{5\times 5}$ represents a convolution operation with a 5×5 filter;
- [x] denotes concatenation.

Finally, a block that consists of a succession of layers was added. These layers are a separable convolution, batch normalization, GlobalMaxPooling2D, rectified linear unit activation, dropout, and a fully connected layer adapting the output to a binary classification. A softmax function is used as a classifier at the end of the architecture.

### 2.5. Model Training and Loss Function

According to our experiment, focal loss [29] better helped diminish the impact of data imbalance compared to the categorical cross-entropy loss. Focal loss has a hyper-parameter called γ to penalize misclassified examples. Our dataset was imbalanced, with a higher prevalence of Bethesda II samples. Therefore, we computed the focal loss with a high γ parameter and assigned automatic loss weights based on the sample distribution of classes. This approach helps mitigate the data imbalance problem and improves model performance with only a minimal increase in computational cost. Non-pretrained weights were initialized using He normal initialization [30], involving sampling from a truncated normal distribution centered around zero. The optimization process employed the adaptive moment estimation (ADAM) algorithm with a learning rate of 0.0001 to oversee model training and fine-tune its weights. The training utilized a mini-batch size of 8 until reaching convergence, determined by identifying optimal parameters through configurations and experiments. Two well-established techniques were adopted to avoid over-fitting in addition to data augmentation. First, the selection of the best model was performed from the validation loss using early stopping with a patience of 20 epochs, and the dropout method was used to reduce over-fitting by intentionally deactivating nodes at every iteration. In all experiments, dropout regularization was applied with a probability of 0.25.

### 2.6. Reliability of the Classification and Explainability

The reliability of the binary classification is provided via a score expressed in percentage from 0% to 100%. This score corresponds to the output of the softmax function, which is analogous to the probability of each image belonging to a class. The FNA-required probability estimates the likelihood that a thyroid nodule needs fine needle aspiration. A threshold probability is set to determine if the model’s prediction indicates the need for FNA.

In our proposed method, the gradient-weighted class activation map (Grad-CAM) technique was used to offer valuable insights into the provided classification. For a given class *c*, the importance weight $\alpha_{k}^{c}$ for the feature maps *k* is computed as $\alpha_{k}^{c}=\frac{1}{Z}\sum_{i}\sum_{j}\frac{\partial y^{c}}{\partial A_{ij}^{k}}$, where $y^{c}$ is the score for class *c*, $A_{ij}^{k}$ is the activation at position (i,j) in feature map *k*, and *Z* is the number of pixels in the feature map. The final Grad-CAM heatmap is given by $L_{\mathrm{Grad}-\mathrm{CAM}}^{c}=\mathrm{ReLU}\left(\sum_{k}\alpha_{k}^{c}A^{k}\right)$ [31].Grad-CAM operates by tracing back through the gradients to generate a coarse localization heat map that pinpoints the most significant regions for the network within the input image. This visualization aids in understanding which parts of the image are most influential in making the classification decision. The prediction of the AI model, in conjunction with the Grad-CAM overlay on the nodule of the original image, provides clinicians with reliable information about the need for FNA for thyroid nodules from US images.

### 2.7. Evaluation

Several common metrics were used to evaluate the performance of our method. These metrics are accuracy, sensitivity, F1-score, and the results of the confusion matrix. Despite its widespread usability, accuracy is not always the most appropriate performance metric, especially when target classes are unbalanced in the dataset [32]. The F1-score takes both precision and recall into consideration in order to evaluate the overall performance of a classification model [33]. It is particularly useful when the dataset is imbalanced, meaning that one class is much more prevalent than the other. The F1-score formula is defined as:

(3) $$F1=2\times \frac{\mathrm{precision}\times \mathrm{recall}}{\mathrm{precision}+\mathrm{recall}}$$

where:

- Precision is the ratio of true positive results to the sum of true positive and false positive;
- Recall is the ratio of true positive results to the sum of true positive and false negative.

We used the floating point operations (FLOPs) metric to measure the computational complexity of our deep learning models. FLOPs represent the number of floating-point operations required for a forward pass through the network. This metric is crucial for evaluating the computational requirements and efficiency of different models.

## 3. Results

**Figure 5:**
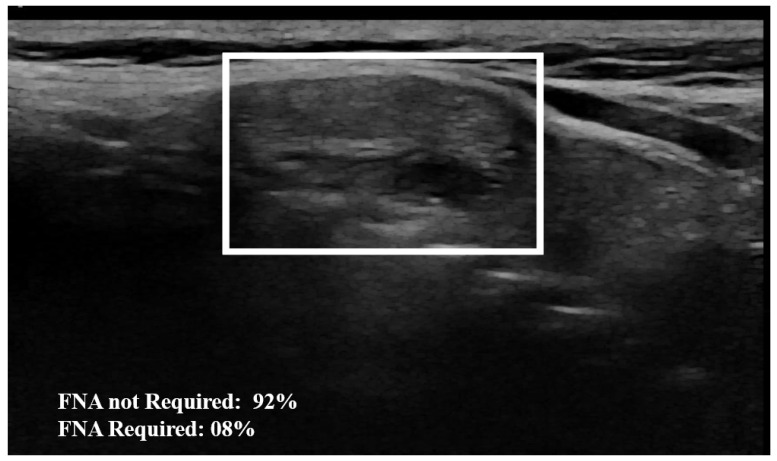
Example of nodule classification: The AI model establishes a 92% probability from the image that the nodule does not require FNA, indicating that FNA is not necessary. The surrounding rectangle indicates the location of the nodule.

**Figure 6:**
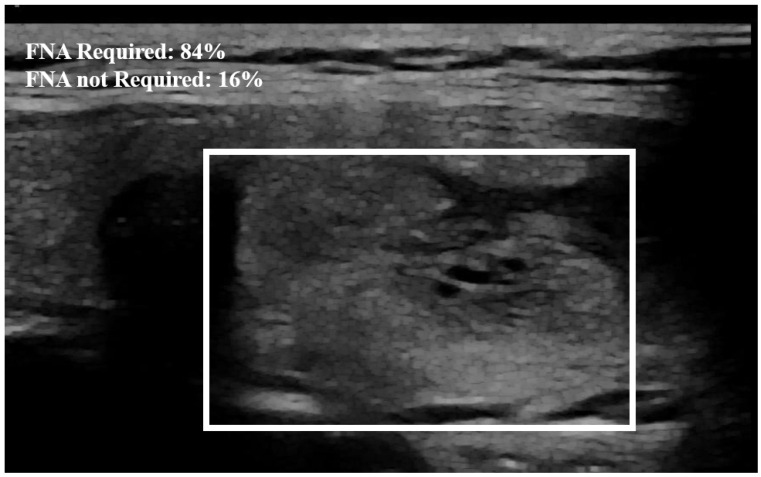
Example of nodule classification. The probability established by the model from the image that the nodule needs FNA is equal to 84%. The surrounding rectangle indicates the location of the nodule.

Examples of our method for the classification of the thyroid nodule images are shown in Figure 5 and Figure 6. The results of the proposed model with and without data augmentation are shown in Table 2. We can conclude that adding synthesized images improved the performance of the model quite effectively. We compared our proposed method with different deep learning architectures (Table 3). We began with experiments using a simple CNN and then fine-tuned pre-trained features to capture specific patterns relevant to our task. This approach led to improved performance compared to training a model from scratch. We conducted numerous experiments on the combination of focal loss parameters (α and γ) and different deep learning architectures. For our classification, α was set to 0.25 for the negative class and 0.75 for the positive class, depending on the class imbalance. Typical values for γ were in the range [0,5]. We experimented with several values of γ within this range for different models listed in Table 3, and γ=3.5 was empirically found to be the optimal value.

**Figure 7:**
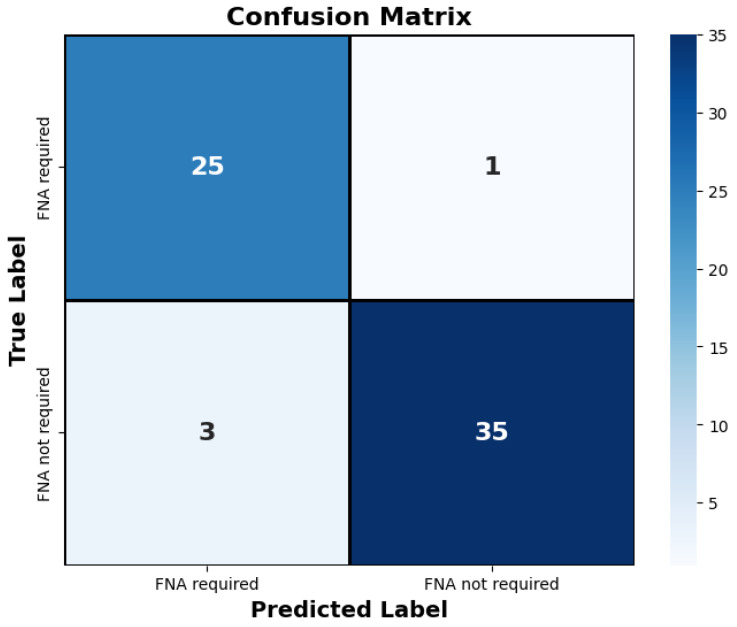
The confusion matrix depicts the proposed approach’s classification of thyroid nodules on the test set. Only four images were misclassified by the model. “FNA required” denotes the necessity for fine needle aspiration for the respective nodule, while “FNA not required” indicates that fine needle aspiration is not necessary.

The proposed method outperformed the other classic approaches. This is due to the fact that the incorporated attention modules are playing an efficient role in extracting the most important features. The other models did not perform well compared to the proposed approach, most likely due to over-fitting. We observed that the other models ended up memorizing some abstract patterns and were highly sensitive to random fluctuations. Hence, the lowest F1-score on this classification task was 0.79, obtained from the predictions done by the simple CNN network. The proposed method only misclassified 4 images out of the 64 test set images, as illustrated by the confusion matrix in Figure 7. During the training session of the model, 20% of the dataset is automatically split for the validation process. We also assessed the model’s generalization capacity on unseen data. In the test set dataset, the model maintained high scores, achieving an average accuracy of 0.94 and a F1-score of 0.93. These results indicate the robustness and effectiveness of the model’s performance in accurately classifying thyroid nodules. The model’s performance evaluation was conducted on individual image database centers to assess the portability of our approach. The average accuracy and F1-score for the test set from the Hospital of Bastia database were 0.87 and 0.89, respectively. Similarly, for the test set from the Hospital of Dijon database, the average accuracy and F1-score were 0.90 and 0.86, respectively. However, the mixed test set from both centers achieved the highest performance, with an F-score of 0.93, as shown in Table 3. We also compared the proposed method with previous studies, as detailed in Table 4. The results demonstrate that our approach provides competitive performance in the classification of thyroid nodules, achieving high accuracy and reliability compared to other existing methods. For instance, in Figure 6, the probability of the nodule requiring FNA based on the model output is established to be 84%. Then, according to our model, this nodule is at high-risk level and fine needle aspiration is recommended.

**Figure 8:**
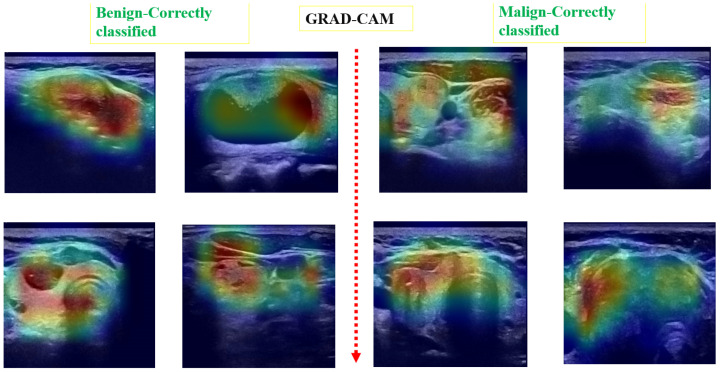
Examples of predicted images with Grad-CAM overlaid on the initial US image. The heatmap highlights regions in the original images that were important for the model’s prediction.

**Figure 9:**
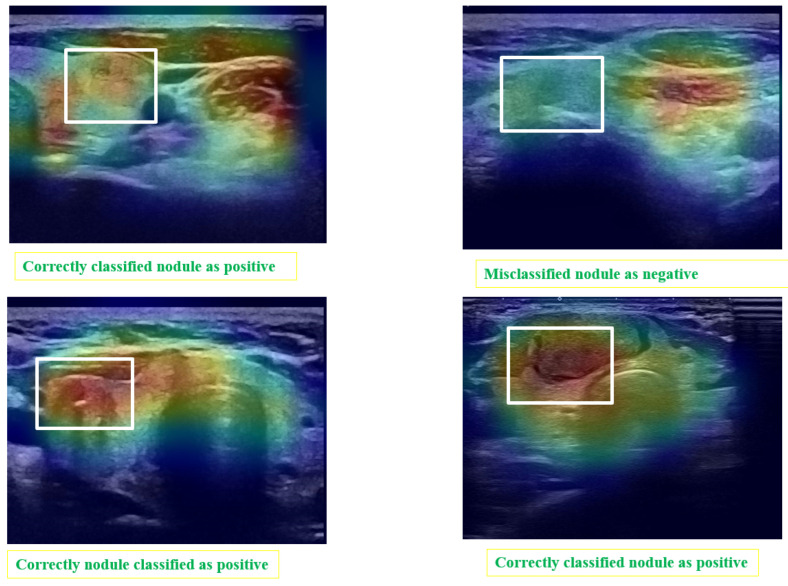
Matching of the Grad-CAM maps and the localization of the module. The areas surrounded by white rectangles indicate the location of nodules identified by experts. Grad-CAM localizes the areas considered by the automatic system as regions of interest.

Post hoc explainability was implemented to illustrate how our artificial intelligence made decisions in classifying nodules, as shown in Figure 8 and Figure 9. The Grad-CAM overlay is applied to the original ultrasound image, revealing that the model primarily focused on information extracted from the nodules’ primary regions. In instances of incorrect predictions, the model’s decisions were influenced by irrelevant parts of the images, as indicated by the absence of a strong heatmap correlation (Figure 9). This misalignment suggests areas where the model’s attention may have been misguided. Additionally, the model sometimes relied on minimal information to classify images. The relevance of Grad-CAM was assessed by sonographers, who found that the bounded regions in Figure 9 align with the relevant locations of the nodules. Conversely, in misclassified images, Grad-cam highlighted irrelevant areas.

The decision of the AI model was compared with the Bethesda classification system and EU-TIRADS standards on the entire testing dataset (Table 5 and Table 6). The model made four mistakes, highlighted in red in the table. Additionally, as illustrated in Figure 7, out of the 64 cases in the test set, only 4 were misclassified. The proposed AI model automatically classifies thyroid nodules with an accuracy performance of 0.94.

## 4. Discussion

The primary aim of this study is to automatically classify thyroid nodules from ultrasound (US) images to guide fine needle aspirations. Our research introduces a novel approach by integrating dual attention modules—channel attention (CAM) and spatial attention (SAM)—within the DenseNet121 architecture to enhance nodule classification. Unlike existing methods that use attention mechanisms to extract features, our method uniquely refines intermediate features through a sequential application of CAM and SAM. This dual attention strategy significantly improves the network’s ability to focus on crucial intermediate features while filtering out irrelevant information, thereby optimizing classification performance. We conducted extensive experiments to compare our proposed method with various deep learning architectures, including simple CNNs, VGG16, EfficientNet-B0, ResNet18, InceptionV3, and DenseNet121. Our method consistently outperformed these models, achieving an average accuracy of 0.94 and an F1-score of 0.93 on the test set. These results showcase its robustness and effectiveness in accurately classifying thyroid nodules. When compared to similar previous studies, our method delivers competitive outcomes for thyroid nodule classification tasks. Although the scope of the comparison is quite similar, the methods used in these studies employ different algorithms and datasets. Our approach, achieving an accuracy of 0.94, is on par with the results from previous studies. This positions our method at the forefront in terms of accuracy and sensitivity. We placed significant emphasis on minimizing false negatives, as overlooking necessary fine needle aspiration is clinically unacceptable. Our experiments also demonstrated that using focal loss for data augmentation enhances robustness when training on small datasets. We compared focal loss with binary cross-entropy and observed a 0.07 improvement in accuracy. Additionally, enriching the dataset with augmented samples increased the accuracy from 0.87 to 0.94.

As explained in Section 1, several previous studies have proposed methods for solving the thyroid nodule classification problem. Among the earliest studies, Zhu et al. employed a ResNet18-based network and applied transfer learning to mitigate overfitting, reporting a classification accuracy of approximately 0.84. To address variations in nodule sizes, Chi et al. [10] utilized GoogLeNet, also known as the Inception network. Building on their method, Nguyen et al. [35] achieved a classification accuracy of 0.92. Table 1 summarizes the classification performance of these previous methods compared to our proposed approach. The results in this table demonstrate that our proposed method outperforms previous studies in the classification of thyroid nodules using ultrasound images.

In our study, we used fundamental image processing techniques to remove artifacts from ultrasound images. To ensure vital diagnostic information remained intact expert radiologists reviewed images before and after artifact removal, confirming that key anatomical and pathological features were preserved. Deep learning models were trained on both the original and the artefact-removed images. These methods collectively ensured that our artifact removal process enhanced image quality without compromising critical information.

Our method was limited to classifying thyroid nodules from only US images into two categories (FNA required or not) without involving any clinical information in the process. Indeed, the system has been trained to perform classification tasks based solely on the acquired images. Thus, the proposed method does not simulate the mode of operation of the physician but provides additional information in order to decide whether to perform FNA or not. We acknowledge that our study is limited by its reliance on a relatively small dataset, which may impact the generalizability of the model across a wide range of clinical environments. However, it is important to note that our system is constructed using ultrasound images acquired from two distinct hospitals in France. This inclusion of data from multiple clinical sites helps to mitigate some of the limitations associated with a smaller dataset by introducing variability in imaging conditions and patient demographics. A larger dataset encompassing samples of all Bethesda scores could enable more comprehensive classification by considering each Bethesda score independently. Moreover, a larger dataset could involve a potential avenue for future work, that is to choose one of the centers as the validation cohort and the other one as the training cohort. However, we were unable to conduct such experiments due to the constraints of our limited dataset. Grad-CAM, a post-explainability algorithm, provides valuable insights into how the model distinguishes between classes in thyroid nodule classification, while limited to visual heatmaps, this method can enhance confidence in deep learning predictions, alleviating concerns about black box models. However, it is important to note that the Grad-CAM algorithm localizes a region of interest, which may not always coincide exactly with the nodule. This suggests that there could be crucial information not visible to the naked eye but essential for making classification decisions. Insufficient training data can lead to Grad-CAM highlighting areas other than the nodule region, underscoring the need for robust and comprehensive datasets to ensure accurate localization and interpretation of relevant features. We have actively involved radiologists throughout the development of our proposed system. Their participation has been essential in ensuring that our system is not only user-friendly but also effectively meets the practical demands of clinical workflows. By incorporating their expertise and feedback at every stage, we have aimed to create a solution that seamlessly integrates into the daily practices of medical professionals, enhancing both usability and relevance in real-world applications. Radiologists and sonographers have guided us on data privacy and regulatory issues, ensuring our model meets high standards of safety and compliance, while their expertise has shaped a secure system for clinical use, further work is needed for integration. This includes obtaining regulatory approvals, ensuring compatibility with existing healthcare systems, and conducting thorough validation to meet clinical standards. The limitations of the proposed network include the lack of discriminative feature extraction using attention mechanisms and reliance on traditional data augmentation techniques. To address these challenges, incorporating generative adversarial networks (GANs) to generate synthetic images could significantly enhance the performance of our system. GANs can provide additional realistic training data, thereby improving the model’s ability to generalize across diverse scenarios. Furthermore, exploring additional processes, such as nodule segmentation and incorporating clinical information, could further enhance the performance of the CAD system. Additionally, expanding the dataset and incorporating the latest deep learning models, such as vision transformers (ViTs), could improve the model’s robustness. However, unlike CNNs, which benefit from inherent inductive biases that aid generalization from smaller datasets, ViTs typically require larger amounts of data to achieve optimal performance.

Future improvements to the proposed method could include integrating clinical data, such as patient history and genetic markers, to enhance classification accuracy. Expanding the dataset by incorporating more samples from several different hospitals and generating realistic synthetic images from these samples are promising avenues for boosting performance. Additionally, incorporating a wider variety of nodule types and demographic features into our CAD system could better represent the diversity found in clinical settings worldwide.

## 5. Conclusions

In conclusion, our deep learning CAD system offers promise for automatically classifying thyroid nodules from US images as requiring FNA or not, with the goal of reducing unnecessary FNAs. The DenseNet-based network with attention modules achieved encouraging results, suggesting its potential to minimize false negatives. Further efforts are needed to entirely eliminate false negatives, enhancing the model’s clinical utility. Grad-CAM heatmaps provide transparency in model decision-making, addressing concerns about neural network opacity. This CAD algorithm can mitigate inter-reader variability and subjectivity in thyroid exam classification, even within standard interpretation criteria like EU-TIRADS.

## Figures and Tables

**Table 1:** Summary of dataset according to the Bethesda category. Bethesda Cat: Bethesda Category.

Bethesda Cat.	Number of Cases	Mean Age	Males/Females	TIRADS [3/4/5]
II	393	43±2	172/221	[167/148/78]
III	98	49±1	43/59	[35/36/27]
IV	56	52±4	21/35	[23/17/16]
V	37	55±3	11/26	[12/16/9]
VI	7	57±1	3/4	[2/1/4]

**Table 2:** Impact of the data augmentation on the classification results. The best results are in bold.

Metrics	No Data Augmentation	Data Augmentation
Accuracy	0.87	**0.94**
F1-score	0.85	**0.93**
Sensitivity	0.88	**0.96**

**Table 3:** Accuracy, F1-score, training time, inference time, number of parameters, and FLOPs of different networks for thyroid nodule classification. The best results are in bold. Acc: accuracy, h: hours, min: minutes, Train.Time: training time, Infer.Time: inference time, Para: parameters, FLOPs: floating point operations (per forward pass), ms: milliseconds.

Methods	Acc	F1-Score	Train.Time	Infer.Time	Para.	FLOPs
Simple CNN [34]	0.76	0.79	8 h:30 min	25 ms/Image	15.40 × $10^{7}$	≈$4.45\times 10^{9}$
VGG16 [22]	0.81	0.84	3 h	15 ms/Image	0.45 × $10^{6}$	≈$11.90\times 10^{9}$
ResNet18 [20]	0.85	0.87	3 h:15 min	20 ms/Image	0.65 × $10^{6}$	≈$1.90\times 10^{9}$
InceptionV3 [23]	0.88	0.92	4 h:25 min	55 ms/Image	12.53 × $10^{6}$	≈$6.70\times 10^{9}$
EfficientNet-B0 [21]	0.83	0.87	5 h:40 min	50 ms/Image	2.25 × $10^{6}$	≈$1.39\times 10^{9}$
DenseNet121 [24]	0.89	0.88	6 h	30 ms/Image	7.1 × $10^{6}$	≈$3.30\times 10^{9}$
**Proposed Method**	**0.94**	**0.93**	**6 h:30 min**	**30 ms/Image**	**7.5 × $10^{6}$**	**≈$3.47\times 10^{9}$**

**Table 4:** Comparison of different CAD systems in classifying thyroid nodule images. RBF: Radial Basis Function; SVM: Support Vector Machine; CBAM: Convolutional Block Attention Module; Acc.: Accuracy; Sen.: Sensitivity.

Methods	Learning Tech	Acc.	Sen.	Metric	Objective
Buda et al. [7]	Faster R-CNN	0.58	0.87	TIRADS	Recommending biopsy
Zhu et al. [12]	ResNet-18	0.93	0.93	TIRADS	Benign vs. Malignant
Wu et al. [14]	(RBF)–NN	0.84	0.92	TIRADS	Benign vs. Malignant
Nguyen et al. [35]	Multi-CNN	0.92	0.95	TIRADS	Benign vs. Malignant
Peng et al. [9]	SVM (Kernel=RBF)	0.88	0.82	Raw images	Benign vs. Malignant
Koh et al. [36]	ResNetV2	0.76	0.91	TIRADS	Benign vs. Malignant
Li et al. [36]	CNN-F	0.85	0.91	TIRADS	Background vs. Benign vs. Malignant
Kwon et al. [15]	VGG16	0.81	0.92	TIRADS	Benign vs. Thyroid Carcinoma
Alyas et al. [37]	RF	0.91	0.94	Raw images	Negative vs. Positive
**Proposed method**	**DenseNet with CBAM**	0.94	0.96	Bethesda	FNA required vs. FNA not

**Table 5:** Classification of nodules using Bethesda system, EU TIRADS score, and Artificial intelligence model for 50 patients, FNA: fine needle aspiration; Red entries under the Clas. of AI Model column indicate incorrect decisions made by the model; Bethesda Std: Bethesda standard; AI: Artificial intelligence.

Patient No	Bethesda Std	EU TIRADS Score	Classification from AI Model
Patient 1	IV	**4**	FNA required
Patient 2	II	**5**	FNA not required
Patient 3	II	**3**	FNA not required
Patient 4	III	**5**	FNA required
Patient 5	IV	**4**	FNA required
Patient 6	II	**3**	FNA not required
Patient 7	V	**5**	FNA required
Patient 8	III	**5**	FNA required
Patient 9	II	**4**	FNA not required
Patient 10	III	**4**	FNA required
Patient 11	II	**3**	FNA required
Patient 12	IV	**5**	FNA required
Patient 13	II	**3**	FNA not required
Patient 14	VI	**5**	FNA required
Patient 15	II	**4**	FNA not required
Patient 16	IV	**3**	FNA required
Patient 17	V	**3**	FNA not required
Patient 18	III	**5**	FNA required
Patient 19	II	**3**	FNA required
Patient 20	IV	**4**	FNA required
Patient 21	II	**4**	FNA not required
Patient 22	VI	**5**	FNA required
Patient 23	II	**4**	FNA not required
Patient 24	V	**4**	FNA required
Patient 25	II	**4**	FNA required
Patient 26	IV	**3**	FNA required
Patient 27	V	**5**	FNA required
Patient 28	VI	**5**	FNA required
Patient 29	IV	**3**	FNA required
Patient 30	III	**4**	FNA required
Patient 31	II	**3**	FNA required
Patient 32	IV	**5**	FNA required
Patient 33	III	**3**	FNA required
Patient 34	IV	**3**	FNA required
Patient 35	V	**4**	FNA required
Patient 36	III	**3**	FNA required
Patient 37	IV	**5**	FNA required
Patient 38	VI	**5**	FNA required
Patient 39	II	**3**	FNA not required
Patient 40	V	**4**	FNA required
Patient 41	II	**3**	FNA not required
Patient 42	II	**5**	FNA not required
Patient 43	IV	**3**	FNA required
Patient 44	II	**5**	FNA not required
Patient 45	IV	**4**	FNA required
Patient 46	II	**3**	FNA not required
Patient 47	VI	**5**	FNA required
Patient 48	III	**4**	FNA required
Patient 49	IV	**3**	FNA required
Patient 50	II	**4**	FNA not required

**Table 6:** Classification of nodules using the Bethesda system, EU TIRADS score, and Artificial intelligence model for 14 patients, FNA: fine needle aspiration; Red entries under the Clas. of AI Model column indicate incorrect decisions made by the model; Bethesda Std: Bethesda standard; AI: Artificial intelligence.

Patient No	Bethesda Std	EU TIRADS Score	Classification from AL Model
Patient 51	IV	**3**	FNA required
Patient 52	III	**4**	FNA required
Patient 53	IV	**3**	FNA required
Patient 54	II	**4**	FNA not required
Patient 55	IV	**3**	FNA required
Patient 56	V	**4**	FNA required
Patient 57	II	**4**	FNA not required
Patient 58	VI	**4**	FNA required
Patient 59	IV	**3**	FNA required
Patient 60	II	**4**	FNA not required
Patient 61	IV	**3**	FNA required
Patient 62	III	**5**	FNA required
Patient 63	IV	**4**	FNA required
Patient 64	V	**4**	FNA required

## Data Availability

Some of the datasets analyzed and used during the present study are publicly available on the kaggle website as explained in Section 2 and the private datasets are available from the corresponding author upon reasonable request.
